# Side-deep slow-release nitrogen fertilization combined with panicle-stage urea topdressing improves energy efficiency, reduces carbon footprint, and enhances economic performance in rice systems

**DOI:** 10.3389/fpls.2026.1962913

**Published:** 2026-09-23

**Authors:** Mingming Hu, Zhixin Li, Yongheng Luo, Kairui Chen, Ying Xiong, Chuanhai Shu, Leilei Li, Biao Li, Zhonglin Wang, Zongkui Chen, Zhiyuan Yang, Yongjian Sun, Jun Ma

**Affiliations:** Rice Research Institute of Sichuan Agricultural University, Sichuan Provincial Key Laboratory of Crop Physiology, Ecology and Cultivation, Chengdu, China

**Keywords:** carbon footprint, economic benefits, energy use efficiency, side-deep placement, slow-release nitrogen fertilizer

## Abstract

**Introduction:**

Although both side-deep placement and slow-release nitrogen fertilizer (SRF) have been widely recognized as effective strategies for improving nitrogen use efficiency in rice, the effects of integrating side-deep slow-release nitrogen fertilization combined with urea topdressing on energy efficiency, carbon footprint (CF), and economic performance remain unclear.

**Methods:**

In this study, seven N treatments were established: no N (0N), traditional fertilization (CK), side-deep placed urea (DU), side-deep placed SRF (DSU), and DSU supplemented with 30% urea at basal, tillering, or panicle stages (DSU+U, DSU+TU, DSU+PU). We systematically evaluated the combined effects of integrating side-deep slow-release nitrogen fertilization combined with urea topdressing on rice productivity, energy input–output structure, CF, and economic performance.

**Results:**

Compared with CK, side-deep placed SRF treatments significantly increased rice yield by 10.65%–23.54%. Under similar total energy inputs, side-deep placed SRF significantly enhanced energy output and net energy, with energy use efficiency (EUE), energy productivity (EP), and energy profitability (EPF) increasing by 9.62%–22.40%, 8.88%–22.86%, and 10.34%–24.07%, respectively, while specific energy (SE) was reduced. Although side-deep placed SRF increased indirect greenhouse gas emissions, substantial reductions in field CH_4_ and N_2_O emissions led to significant decreases in CF by 30.69%–41.33%. Meanwhile, yield gains effectively offset higher production costs, resulting in a 22.57%–76.78% increase in net ecosystem economic benefit (NEEB). Among all treatments, DSU+PU achieved the highest yield, energy efficiency, and economic returns while maintaining the lowest CF.

**Discussion:**

Overall, DSU+PU represents a practical cleaner-production strategy that simultaneously enhances energy use efficiency, reduces carbon footprint, and improves economic viability, providing an effective pathway toward sustainable rice production.

## Highlights

- Slow-release N fertilizer (SRF) enhanced energy use efficiency.
- SRF reduced carbon footprint by suppressing CH4 and N2O emissions.
- Yield-driven economic gains offset higher fertilizer costs, resulting in a higher NEEB.
- DSU+PU provides a low-carbon, efficient, and economically feasible nitrogen strategy.

## 1 Introduction

Rice (*Oryza sativa L*.) serves as a primary staple for over half of the world’s population, and ensuring its stable and high yield is essential for food security (Xu et al., 2022a). Nitrogen (N) fertilizer is a critical input for rice production, yet inappropriate application rates, fertilizer types, or application methods often lead to low N use efficiency, resulting in reduced biomass production per unit N input (Hou et al., 2023). This inefficiency exacerbates multiple environmental pressures, including enhanced ammonia losses, increased nitrate leaching, and elevated greenhouse gas emissions (Huo et al., 2026), but also increases energy consumption, labor input, and production costs, ultimately reducing economic returns (Gao et al., 2025). Consequently, identifying N management approaches that balance high productivity, improved energy efficiency, and reduced environmental impacts remains a critical challenge for sustainable rice systems.

In recent years, slow-release nitrogen fertilizer (SRF) are widely regarded as an effective approach for enhancing N use efficiency and reduce N losses due to their prolonged and more stable nutrient release patterns (Zhang et al., 2022). Side-deep placement involves applying fertilizer at a depth of approximately 3–5 cm beneath the soil surface, close to the root zone, thereby effectively reducing ammonia volatilization and improving root nitrogen uptake (Wang et al., 2025). Previous studies have demonstrated that combining these two practices generates significant synergistic effects; for example, compared with traditional surface broadcasting, side-deep placed SRF has been reported to increase rice yield by 10%–23% while enhancing agronomic nitrogen use efficiency by 38%–89% (Hu et al., 2025). Moreover, this fertilization strategy can reduce CH_4_ and N_2_O emissions by 25.34% and 48.97%, respectively (Lan et al., 2024). This may be attributed to the fact that side-deep placed SRF provides a more stable N supply, which helps regulate soil N dynamics, reduce excessive dissolved organic carbon (DOC) accumulation, and maintain a relatively higher redox potential (Lan et al., 2024). However, the production of SRF is associated with relatively high energy consumption and carbon emission coefficients, and its market price is substantially higher than that of conventional urea (Liu et al., 2025), leading to ongoing debate over whether SRF truly offers advantages in terms of energy performance and ecosystem economic benefits under practical farming conditions. In addition, relying solely on SRF as a basal application may not adequately match the dynamically changing N demand of rice during its growth period, particularly during the critical stages from tillering to panicle initiation when nitrogen uptake rates increase significantly, potentially leading to a temporary insufficiency in N supply (Ke et al., 2023). Therefore, side-deep slow-release nitrogen fertilization combined with urea topdressing may represent a promising strategy to simultaneously achieve yield enhancement, energy savings, emission reduction, and cost control. Previous studies have shown that the combined application of slow-release fertilizer and conventional urea can better synchronize N supply with crop N demand than the sole application of either fertilizer, and that partial substitution with approximately 30% conventional urea can maintain yield while improving N use efficiency (Lv et al., 2021). Based on these findings, a 30% urea substitution ratio was adopted in the present study. This proportion retained 70% of the total N as SRF to provide sustained N supply, while allocating the remaining 30% as readily available urea to enable N supplementation at different growth stages.

In rice production systems, chemical fertilizers—particularly N fertilizers—constitute the dominant share of energy inputs, contributing over 30%–40% of total energy use (Hosseini et al., 2024). Accordingly, adjustments in nitrogen management practices can exert a direct influence on the energy configuration and overall efficiency of the production system. Previous studies have shown that, compared with conventional fertilization, SRF involve higher energy inputs but can ultimately enhance energy use efficiency and net energy by substantially increasing energy output from rice production (Lan et al., 2024). Similarly, in wheat production, appropriate application of SRF has been reported to increase energy productivity by 3.9%–12.4%, while excessive nitrogen inputs (>300 kg N ha^-1^) lead to a decline in energy productivity (Xu et al., 2022b). Consequently, under reasonable nitrogen input levels, systematically evaluating the energy performance of different nitrogen management strategies from an energy input–output perspective is essential for developing efficient and low-energy rice production systems.

Meanwhile, carbon footprint (CF) are increasingly used as key sustainability metrics to evaluate the environmental impacts associated with agricultural production. CF represents the total greenhouse gas emissions generated from both on-field processes and the production of agricultural inputs throughout crop production (Gao et al., 2023). Earlier studies have indicated that changes in fertilization practices or fertilizer types can significantly affect crop CF, thereby altering the environmental costs of agricultural production systems (Lan et al., 2024). In addition, economic performance is a key determinant for the adoption and large-scale implementation of agricultural technologies. Although SRF can increase yield and reduce labor inputs, their material costs are relatively high (Gao et al., 2024), and staged urea application may modify input structures and ultimately influence economic returns. However, comprehensive evaluations of the combined effects of side-deep placed SRF with staged urea application on energy input–output, CF and economic performance remain limited.

Consequently, a three-year field experiment (2023–2025) was carried out in a representative rice-growing area of Southwest China, involving seven nitrogen treatments: no N (0N), conventional fertilization (CK), side-deep placed urea (DU), side-deep placed slow-release N fertilizer (DSU), and three DSU-based treatments supplemented with 30% urea applied at the basal, tillering, or panicle stage (DSU+U, DSU+TU, DSU+PU). We hypothesized that integrating side-deep placed SRF with staged urea application (with partial substitution, e.g., 30% urea) would improve the synchronization between N supply and crop demand, thereby enhancing rice yield, energy use efficiency, and economic returns, while reducing CF, ultimately improving the overall sustainability of rice production systems. This study aimed to: (1) comprehensively assess agricultural energy inputs, energy outputs, and energy use efficiency under different N management modes; (2) examine the impacts of these strategies on greenhouse gas emissions as well as carbon footprint; (3) compare cost structures, net returns, and net ecosystem economic benefits (NEEB) among treatments; and (4) identify the optimal N management strategy for mechanized transplanted rice systems, aiming to enhance yield, improve energy use efficiency, reduce greenhouse gas emissions, and increase economic benefits. By jointly evaluating yield, energy use, environmental impacts, and economic outcomes, this work contributes scientific evidence and actionable insights to support optimized nitrogen management, enhance agricultural energy efficiency, and facilitate the development of greener and lower-carbon rice production systems.

## 2 Materials and methods

### 2.1 Experimental site and meteorological conditions

Field trials were conducted from 2023 to 2025 at the Chongzhou experimental station of Sichuan Agricultural University, Sichuan, China (30°42′ N, 103°28′ E). The location lies in a subtropical monsoon region at an elevation of about 352 m, and associated meteorological conditions are shown in **Supplementary Figure S1**. The topsoil (0–20 cm) has a sandy loam texture, and its initial physicochemical characteristics are summarized in **Supplementary Table S1**. The three-year experiments were conducted in the same experimental field, with no preceding crop grown before rice cultivation in each year. Before each rice-growing season, the field was tilled and re-divided into experimental plots, and the N treatments were re-randomized among plots.

### 2.2 Experimental design

The experiment followed a randomized complete block design with a single nitrogen factor, comprising seven treatments: no nitrogen input (0N), traditional fertilization (CK), side-deep placed urea (DU), side-deep placed SRF (DSU), and three DSU-based treatments supplemented with 30% urea applied at the basal (DSU+U), tillering (DSU+TU), and panicle stages (DSU+PU). All N fertilizer treatments received an equal total nitrogen rate of 150 kg ha^-1^, with fertilization details summarized in **Table 1**. For the 0N and CK treatments, the fertilizer outlet of the transplanter was closed, and only machine transplanting was performed. For the remaining treatments, basal N fertilizer was applied simultaneously with machine transplanting using a side-deep fertilization device. The fertilizer was placed in a continuous band approximately 3–5 cm below the soil surface and 2–5 cm horizontally from the rice seedlings. Tillering N was manually broadcast at 10 days after transplanting. Panicle N was manually broadcast at the fourth-leaf-from-the-top stage, corresponding to approximately 65 days after transplanting during panicle-development stage. The actual application dates were June 30, June 24, and June 24 in 2023, 2024, and 2025, respectively.

**Table 1:** Experiment treatments (kg ha^-1^).

Treatment	Basal N rate		Tillering N rate (urea)	Panicle N rate (urea)	Total N rate
	SRF	Urea			
0N	0	0	0	0	0
CK	0	60	45	45	150
DU	0	150	0	0	150
DSU	150	0	0	0	150
DSU+U	105	45	0	0	150
DSU+TU	105	0	45	0	150
DSU+PU	105	0	0	45	150

The SRF used in this study was a resin-coated product (N ≥ 44.0%) manufactured by Hebei DeWodo Fertilizer Co., Ltd., while urea (N ≥ 46.0%) served as the conventional nitrogen source. Potassium was supplied as potassium chloride (K_2_O ≥ 60%) at a rate of 150 kg ha^-1^, and phosphorus was applied as single superphosphate (P_2_O_5_ ≥ 12%) at 75 kg ha^-1^. Both phosphorus and potassium fertilizers were incorporated once, one day prior to transplanting. The field experiment consisted of plots measuring 19 m^2^, arranged with three replicates per treatment, resulting in a total of 21 plots. Rice seedlings were transplanted at a hill spacing of 30 cm × 18 cm, with 3–5 seedlings per hill. To maintain independent irrigation and drainage among treatments, plots were separated by plastic-covered ridges. Seedlings were raised using blanket nursery trays, with 80 g of dry seeds sown per tray. The rice cultivar used was the widely grown indica hybrid “Chuankangyou 6308”, which is commonly cultivated in Southwest China. Detailed schedules for sowing, transplanting, and harvesting are provided in **Supplementary Table S2**, while field management practices and machinery specifications are summarized in **Supplementary Table S3**. Water management was conducted as follows: the soil was kept moist during transplanting; intermittent irrigation was applied during the tillering stage, followed by mid-season drainage at the end of tillering; from the jointing stage to maturity, intermittent irrigation was continued. Pest, weed, and disease control, as well as other field management practices, were carried out according to local conventional high-yield cultivation practices. The SRF used in this study had a nutrient release period of approximately 90 days. The N release characteristics of SRF under field conditions were determined using the *in situ* buried-bag method. On the day of rice transplanting, 10 g of resin-coated SRF was weighed and placed into nylon mesh bags (15 × 10 cm, 60 mesh). The bags were buried between rice rows at a soil depth of 3–5 cm. Three replicate bags were retrieved at 10-day intervals, with a total of 10 sampling times. After retrieval, the fertilizer samples were carefully cleaned and completely dried in an oven at 40 °C. The cumulative nutrient release rate of SRF was then calculated using the mass-difference method. The resulting release pattern is presented in **Figure 1**. The SRF showed a sustained N release pattern that broadly corresponded with rice N demand across key growth stages. During the tillering stage (approximately 10–40 days), SRF maintained substantial N release, supporting vegetative growth. N release continued through the jointing and panicle development stages (approximately 40–80 days) and remained detectable from heading initiation to full heading (approximately 80–100 days), indicating a prolonged N supply into the reproductive stage. Nevertheless, as the N release rate gradually declined during the mid-to-late growth period, supplementary readily available urea at the panicle stage may have better complemented SRF-derived N supply during the period of high N demand for panicle development and subsequent grain formation.

**Figure 1:**
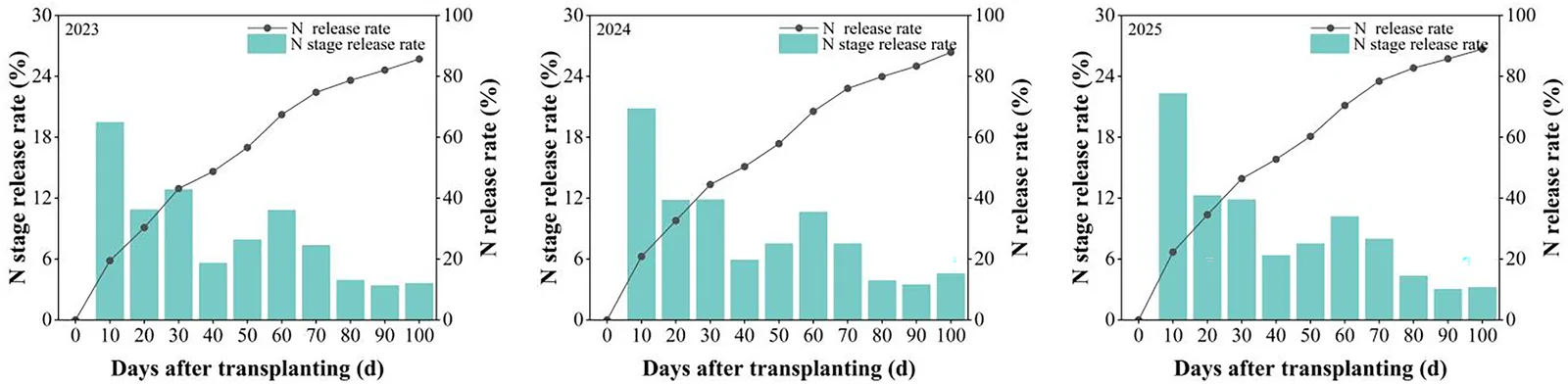
Cumulative nitrogen release curve of the slow-release nitrogen fertilizer determined by the field buried-bag method under field conditions.

### 2.3 Grain and straw yield

Rice was harvested separately from each plot at maturity. Grain moisture content was assessed with a portable analyzer, and yield data were normalized to 13.5% moisture. Straw yield was calculated based on the oven-dried dry weight.

### 2.4 Energy balance

Energy use in rice production can be divided into two major components: energy inputs and energy outputs. Energy inputs mainly comprise machinery operation, fuel, electricity, irrigation water, pesticides, fertilizers, and labor, which were quantified by multiplying the amount of each input by its corresponding energy coefficient. Energy outputs are estimated based on the grain and straw yields obtained at maturity, combined with their respective energy equivalents. At harvest, rice grain and aboveground straw were removed from each experimental plot, and no rice straw was incorporated into the soil during the three-year experiment. The removed straw was considered a recoverable agricultural by-product and was therefore included in the calculation of energy output. The parameters related to machinery inputs are summarized in **Supplementary Table S4**, whereas the energy conversion coefficients for different production inputs and outputs are provided in **Supplementary Table S5**. Estimates of per-hectare resource consumption under each nitrogen treatment are shown in **Supplementary Table S6**. The energy equivalents of machinery-related inputs were calculated as follows (Yuan et al., 2019):


$${\text{Energy}}_{\text{machinery}}=\frac{\text{H}\times 68.4\;({\text{MJ kg}}^{-1})\times \text{W}}{\text{L}\times \text{E}}$$

where, H, W, L and E stand for working hours of machinery in the field (h ha^-1^), weight of machinery (kg), total operational life of machinery (h), and field working efficiency (%), respectively. The total energy input from machinery was calculated as the sum of energy input from plowing machine, harrow, rice transplanter, sprayer, and harvester.

Based on the quantified energy inputs and outputs, a set of energy performance indicators—including net energy, energy use efficiency, specific energy, energy productivity, and energy profitability—were derived using the following equations (Yang et al., 2023):


$$\text{Net energy}(\text{NE},{\text{ MJ ha}}^{-1})=\text{Energy output}({\text{MJ ha}}^{-1})–\text{Energy input}({\text{MJ ha}}^{-1})$$

$$\text{Energy use efficiency}(\text{EUE},{\text{ MJ MJ}}^{-1})=\text{Energy output}({\text{MJ ha}}^{-1})/\text{Energy input}({\text{MJ ha}}^{-1})$$

$$\text{Specific energy}(\text{SE},{\text{ MJ kg}}^{-1})=\text{Energy input}({\text{MJ ha}}^{-1})/\text{Rice yield}({\text{kg ha}}^{-1})$$

$$\text{Energy productivity}(\text{EP},{\text{ kg MJ}}^{-1})=\text{Rice yield}({\text{kg ha}}^{-1})/\text{Energy input}({\text{MJ ha}}^{-1})$$

$$\text{Energy profitability}(\text{EPF},{\text{ MJ MJ}}^{-1})=\text{Net energy}({\text{MJ ha}}^{-1})/\text{Energy input}({\text{MJ ha}}^{-1})$$

### 2.5 CH_4_ and N_2_O emissions

CH_4_ and N_2_O emissions were monitored during all three rice-growing seasons (2023, 2024, and 2025) using the static chamber–gas chromatography method. Gas sampling was conducted between 09:00 and 11:00 a.m. Gas samples (20 mL) were collected from the chamber headspace at 0, 10, 20, and 30 min after chamber closure using a disposable syringe, and subsequently analyzed in the laboratory. Meanwhile, the air temperature inside the chamber and chamber height were recorded simultaneously. Gas sampling commenced on the first day after rice transplanting. Following fertilization events, samples were collected on days 1, 4, and 7, and thereafter at 7-day intervals until harvest. CH_4_ and N_2_O fluxes were calculated using the following equation (Lan et al., 2024):


$$\text{F}=\text{ρ}\times \text{h}\times \frac{\text{dC}}{\text{dt}}\times \frac{273}{273+T}\times 60$$

where, F denotes the gas flux (mg m^-2^ h^-1^); ρ refers to the gas density of CH_4_ and N_2_O under standard conditions (0.714 and 1.964 kg m^-3^, respectively); h represents the chamber height (m). The term $\frac{\text{dC}}{\text{dt}}$ indicates the rate of change in gas concentration over time, which was obtained by linear regression based on four consecutive concentration measurements. T is the mean air temperature inside the chamber (°C), and the constant 60 was used to convert the time unit from minutes to hours.

Cumulative gas emissions were estimated using the equation below (Lan et al., 2024):


$$\text{f}=\sum_{i}^{n}(F_{i+1}F_{i})/2\times (d_{i+1}-d_{i})\times 24\times 10^{-2}$$

Where, f denotes cumulative gas emissions (kg ha^-1^), n is the total number of sampling events, F_i_ and F_i+1_ represent gas fluxes measured at the ith and (i+1)th sampling times, respectively, and d_i_ and d_i+1_ indicate the corresponding sampling dates.

### 2.6 Carbon footprint

CF quantifies greenhouse gas emissions in rice agroecosystems based on a life cycle assessment (LCA) approach. In this study, the system boundary of the LCA mainly included: (1) indirect greenhouse gas emissions associated with agricultural inputs, such as machinery operation, fuel, electricity, pesticides, seeds, fertilizers, and labor; and (2) greenhouse gas emission released directly from rice fields.

CF was calculated using the following equation (Shu et al., 2025):


$$\begin{array}{c} \text{Total GHG emissions}\\ =\sum_{i}^{n}(Al_{i}\times EF_{i})+28\times \text{f}({\text{CH}}_{4})+265\times \text{f}({\text{N}}_{2}\text{O}) \end{array}$$

CF=Total GHG emissions/Grain yield

where CF denotes the yield-scaled carbon footprint (kg CO_2_-eq kg^-1^); total GHG emissions refer to the area-based greenhouse gas emissions from rice production (kg CO_2_-eq ha^-1^). *Al_i_* indicates the quantity of each agricultural input, while *EF_i_* represents the corresponding emission factor for that input (**Supplementary Table S7**). The values of 28 and 265 are the global warming potentials of CH_4_ and N_2_O, respectively, over a 100-year assessment period.

### 2.7 Economic benefits

Economic performance was assessed using net return, benefit-to-cost ratio, and NEEB, which were calculated based on the equations described below. **Supplementary Table S8** summarizes the monetary costs (USD) associated with different production inputs in rice cultivation, as well as the average market price of rice grain used in the analysis.


$$\text{Net return}(\text{USD }\${\text{ ha}}^{-1})=\text{Gross return}(\text{USD}\${\text{ ha}}^{-1})–\text{Production cost}(\text{USD}\${\text{ ha}}^{-1})$$

$$\text{Benefit}-\text{to}-\text{cost ratio}=\text{Net return}(\text{USD }\${\text{ ha}}^{-1})/\text{Production cost}(\text{USD }\${\text{ ha}}^{-1})$$

NEEB=Net return–Total GHG emissions cost

where, the total GHG emissions cost (USD$ ha^-1^) was calculated based on total GHG emissions (kg CO_2_-eq ha^-1^) and the carbon trading price (USD$ 16.4 t^-1^ CO_2_-eq ha^-1^) (Daba et al., 2025).

### 2.8 Determine the optimal nitrogen fertilizer management strategy

To comprehensively evaluate the effects of different treatments on rice yield, energy use efficiency, greenhouse gas emissions, CF and economic benefits, principal component analysis and a membership function approach were employed to identify the optimal nitrogen management strategy.

(1) Principal component analysis (PCA): Principal components were retained according to the Kaiser criterion (eigenvalues > 1), with the selected components explaining more than 85% of the cumulative variance.

(2) Membership function value:


μ(Xi)=(Xi−Xmin)/(Xmax−Xmin), i=1,2,3,…,n,

where Xi represents the i-th integrated indicator, Xmin is the minimum value of that indicator, and Xmax is the maximum value of that indicator.


$$\text{Weight}:{\text{W}}_{\text{i}}={\text{P}}_{i}/\sum_{\text{i}=1}^{\text{n}}\text{Pi}$$

Where W_i_ represents the weight of the i-th principal component, and P_i_ is the contribution rate of the i-th component.

(3) Comprehensive score:


$$\text{D}=\sum_{\text{i}}^{\text{n}}\left[\mu (X\text{i})\times Wi\right]$$

where D denotes the comprehensive evaluation score for each treatment.

### 2.9 Statistical analysis

Prior to statistical analysis, data were tested for normality and homogeneity of variances using the Shapiro–Wilk and Levene’s tests, respectively. Data processing and graphical outputs were generated using Microsoft Excel 2016 (Microsoft, Redmond, WA, USA), SPSS 27.0 (IBM, Armonk, NY, USA), and Origin 2024 (OriginLab, Northampton, MA, USA). Data were analyzed using analysis of variance (ANOVA). For each experimental year, differences among N treatments were assessed separately, and mean comparisons were performed using the least significant difference (LSD) test at P < 0.05. In addition, a combined ANOVA across the three experimental years was conducted, with year (Y) and N treatment (N) treated as fixed factors and block treated as a random factor. The main effects of year and N treatment and their interaction (Y × N) were evaluated, with the corresponding significance levels for all measured variables presented in **Supplementary Table S9**. Three-year means were additionally calculated to characterize the overall treatment performance across the experimental period.

## 3 Results

### 3.1 Rice productivity

Compared with CK, SRF treatments significantly increased grain yield, with a three-year average ranging from 9975.17–11232.74 kg ha^-1^, representing an increase of 10.65%–23.54%, and the highest value was observed under DSU+PU, whereas DU did not differ significantly from CK (**Figure 2A**). A similar trend was found for straw yield: DU increased straw yield by 6.25%, while SRF treatments enhanced it by 10.54%–24.68% (**Figure 2B**). In addition, compared with DSU, shifting 30% of nitrogen to later growth stages led to an overall increase in both grain and straw yields, among which DSU+PU significantly increased these two yield components by 11.65% and 12.79%, respectively.

**Figure 2:**
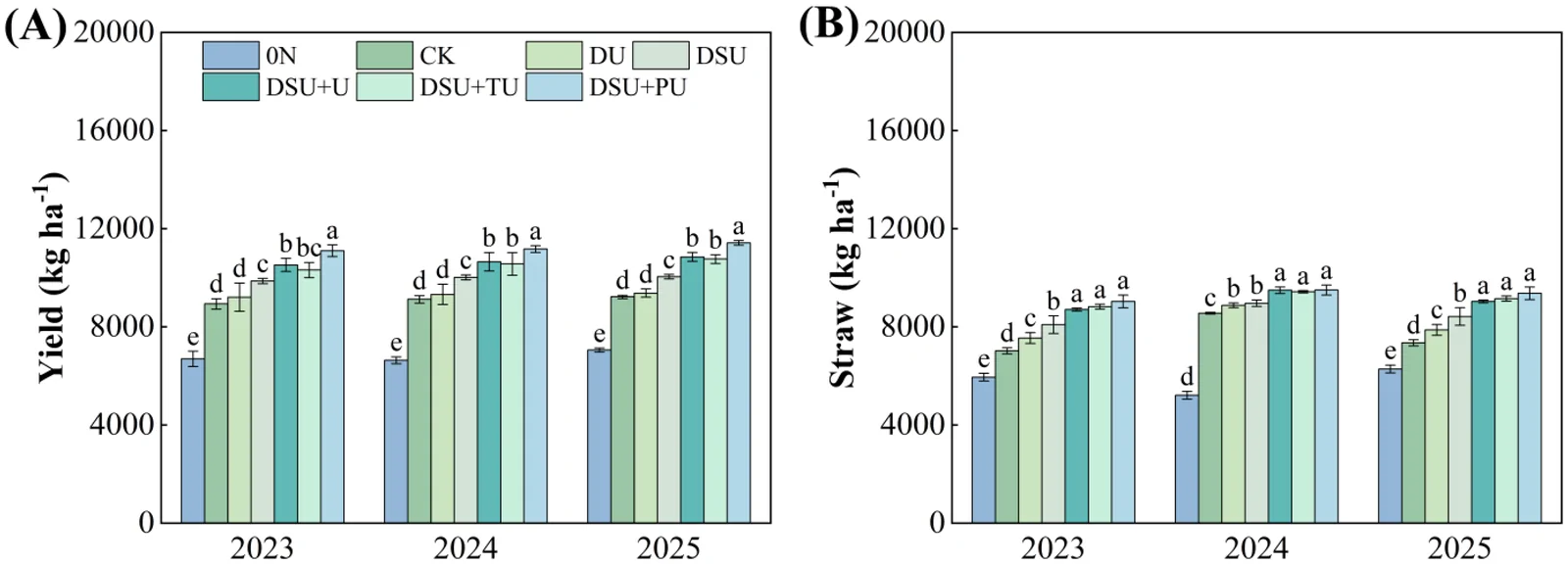
Rice **(A)** and straw **(B)** yields under different nitrogen fertilizer management modes from 2023 to 2025. Different letters indicate significant differences within the same year at *P* < 0.05.

### 3.2 Energy input

2023–2025, the total energy input of rice production showed no substantial differences among nitrogen management practices, but all values were markedly greater than those observed under the 0N treatment (**Figure 3**). Compared with CK, DU reduced total energy input due to lower labor requirements. In contrast, SRF treatments consistently increased total energy consumption, mainly due to the substantially greater energy demand required for producing slow-release fertilizers compared with conventional urea, which resulted in higher fertilizer-related energy inputs and stabilizing total energy input at 26918.69–27628.29 MJ ha^-1^. Moreover, relative to DSU, the co-application treatments slightly reduced total energy input due to partial replacement with 30% urea.

**Figure 3:**
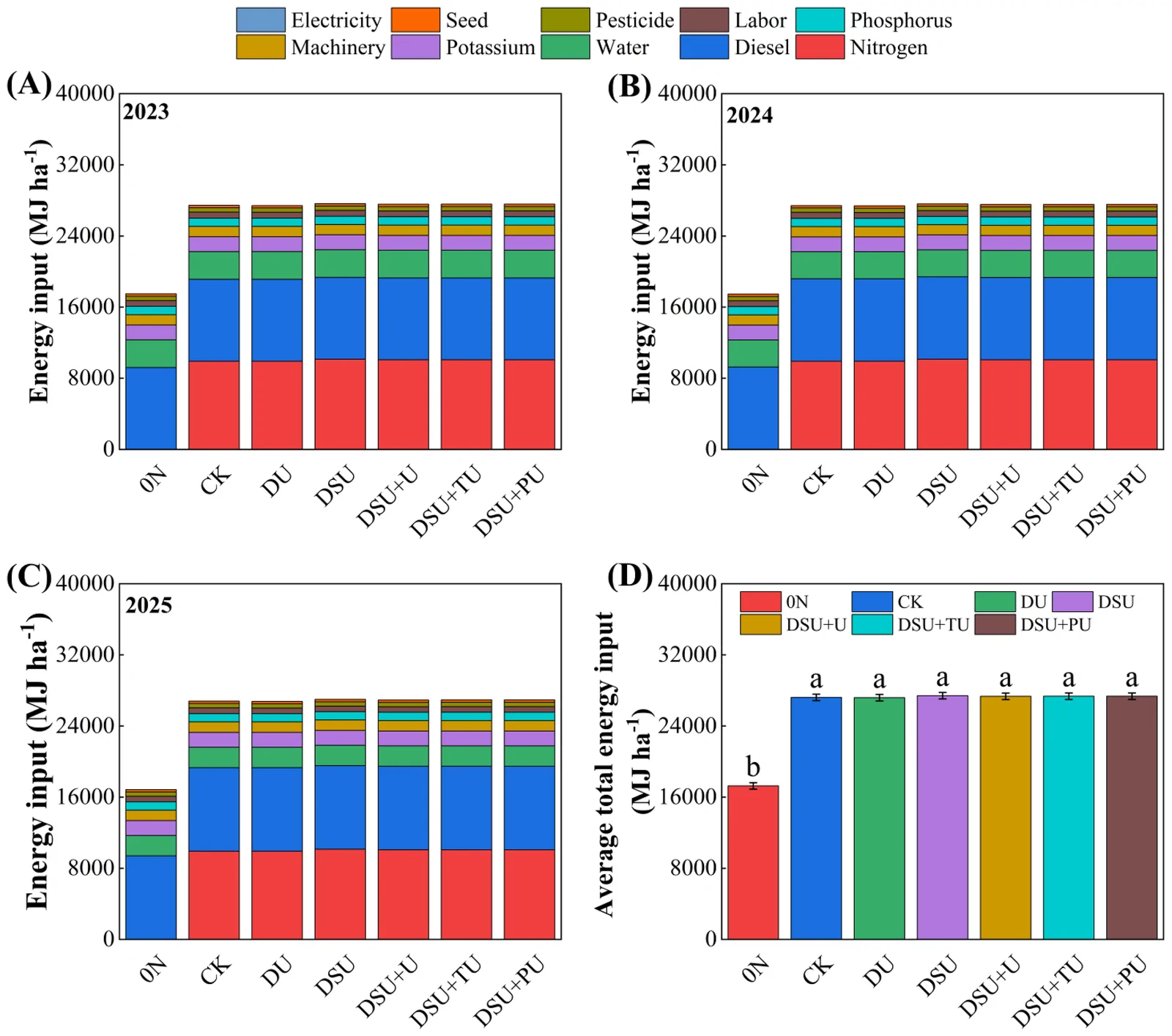
Agricultural energy inputs for rice production under different nitrogen fertilizer management modes from 2023 to 2025. **(A)** Energy input in 2023; **(B)** Energy input in 2024; **(C)** Energy input in 2025; **(D)** Average total energy input from 2023 to 2025.

Across all nitrogen management modes, the relative contributions of different agricultural inputs to total energy consumption followed a consistent ranking: nitrogen fertilizer > diesel > water > potassium fertilizer > machinery > phosphorus fertilizer > labor > pesticides > seeds > electricity (**Supplementary Figure S2**). Among them, nitrogen fertilizer and diesel were the major contributors to energy input, together accounting for over 70% of the total energy consumption. Compared with CK, SRF treatments increased the proportion of fertilizer-related energy input to 36.84%–36.99%, while reducing the shares of other inputs accordingly.

### 3.3 Energy output and use

Rice energy output and energy use efficiency varied significantly among nitrogen management strategies (**Figure 4**). Compared with CK, optimized nitrogen management increased both energy output and net energy. Specifically, DU increased these two indicators by 3.83% and 4.12%, respectively, while SRF treatments resulted in much larger improvements, with 3-year average increases of 10.38%–22.98% and 11.11%–24.68%, respectively, and the greatest enhancement observed in DSU+PU (**Figures 4A, B**). In addition, relative to DSU, postponing 30% urea application further improved energy output and net energy, with DSU+PU increasing by 11.42% and 12.21%, respectively.

**Figure 4:**
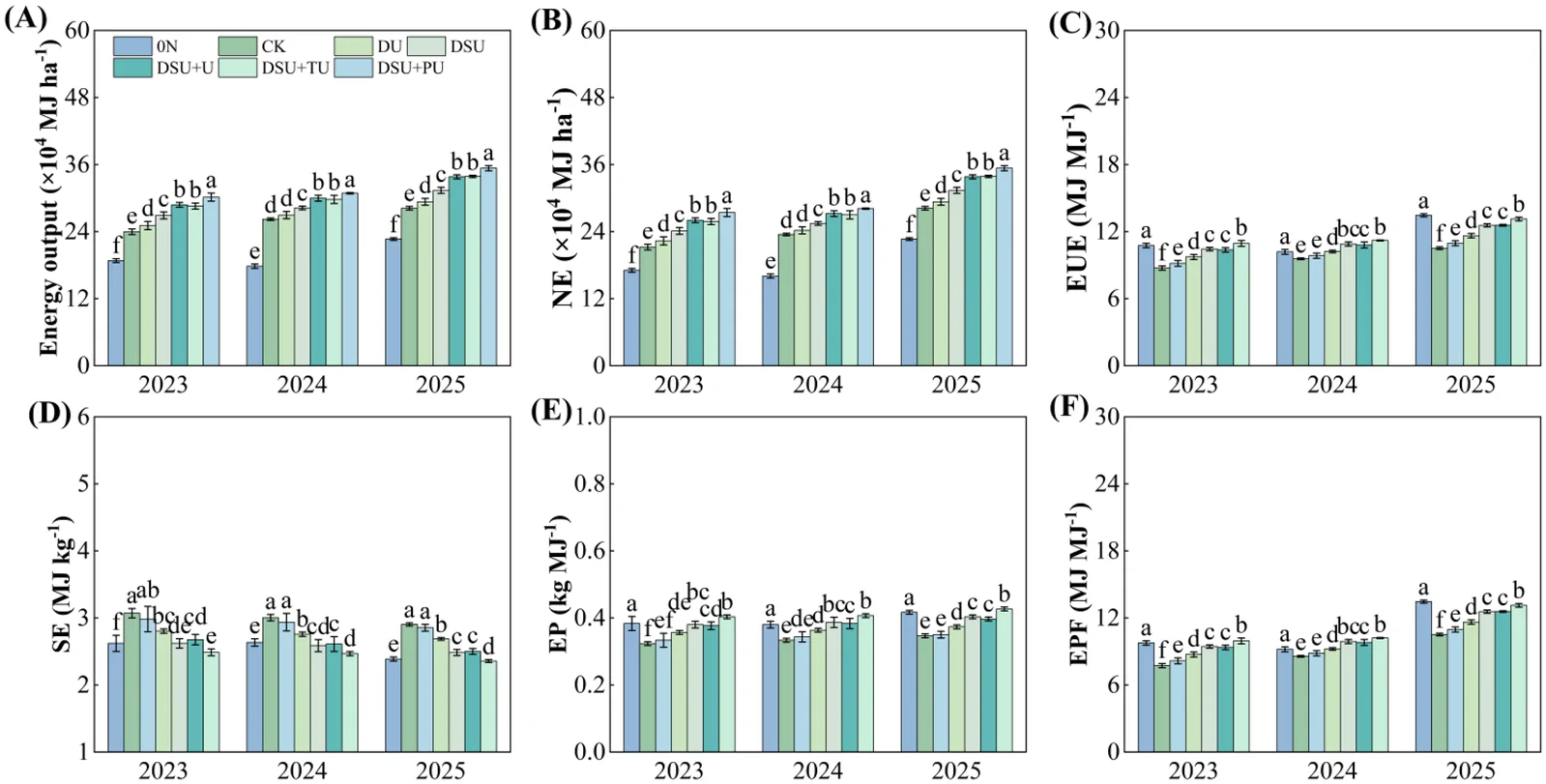
Energy output and utilization in rice production under different nitrogen fertilizer management modes from 2023 to 2025. **(A)** Energy output; **(B)** NE: Net energy; **(C)** EUE: Energy use efficiency; **(D)** SE: Specific energy; **(E)** EP: Energy productivity; **(F)** EPF: Energy profitability. Different letters indicate significant differences within the same year at *P* < 0.05.

Compared with CK, SRF treatments markedly increased EUE, EP, and EPF, with 3-year average improvements of 9.62%–22.40%, 8.88%–22.86%, and 10.34%–24.07%, respectively, again highest under DSU+PU (**Figures 4C, E, F**). Conversely, SRF significantly reduced SE, with a 3-year average decrease of 8.18%–18.61%, reaching the lowest value under DSU+PU (**Figure 4D**). Differences between DU and CK were generally non-significant across all indicators. Furthermore, compared with DSU, postponing nitrogen application increased EUE, EP, and EPF by 11.66%, 13.89%, and 12.47%, respectively, while SE decreased by 11.27% under DSU+PU.

### 3.4 CF

From 2023 to 2025, indirect GHG emissions from rice production showed significant variation among nitrogen management regimes and were consistently higher than those observed under the 0N treatment (**Figures 5A–D**). Compared with CK, SRF treatments significantly increased indirect GHG emissions, primarily due to the higher emission coefficients associated with the production of SRF, however, partial substitution with urea slightly mitigated this increase. The seasonal dynamics of CH_4_ and N_2_O emission fluxes during the three rice-growing seasons are shown in **Supplementary Figures S3**, **S4**, respectively. Both CH_4_ and N_2_O fluxes exhibited pronounced temporal variations during the rice-growing seasons, although their emission patterns and magnitudes varied among years and nitrogen management treatments. In terms of seasonal cumulative emissions, optimized nitrogen management markedly reduced paddy field CH_4_ and N_2_O emissions (**Figures 5E–G**). Compared with CK, SRF treatments reduced cumulative CH_4_ and N_2_O emissions by 23.25%–35.84% and 24.38%–40.27%, respectively, with the lowest values observed under DSU, followed by DSU+PU.

**Figure 5:**
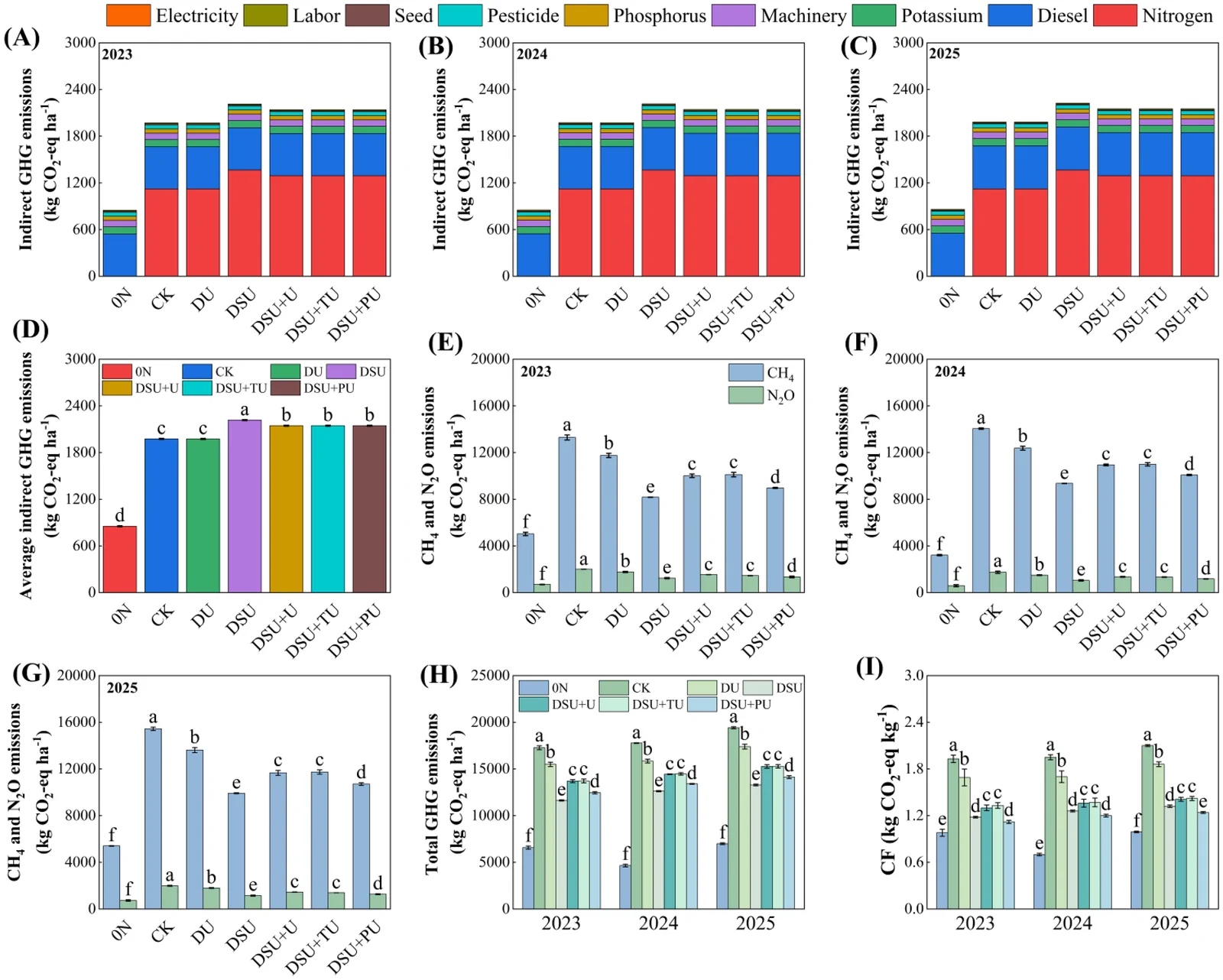
Carbon footprint in rice production under different nitrogen fertilizer management modes from 2023 to 2025. Different letters indicate significant differences within the same year at *P* < 0.05. **(A)** Indirect GHG emissions in 2023; **(B)** Indirect GHG emissions in 2024; **(C)** Indirect GHG emissions in 2025; **(D)** Average indirect GHG emissions from 2023 to 2025; **(E)** CH_4_ and N_2_O emissions in 2023; **(F)** CH_4_ and N_2_O emissions in 2024; **(G)** CH_4_ and N_2_O emissions in 2025; **(H)** Total GHG emissions; **(I)** CF: Carbon footprint.

When indirect and field emissions were considered together, SRF treatments significantly reduced total GHG emissions from rice production, with 3-year average reductions of 20.19%–31.07% (**Figure 5H**). Furthermore, SRF treatments significantly decreased CF by 30.69%–41.33%, with the lowest CF recorded under DSU+PU (**Figure 5I**).

In terms of emission composition, CH_4_ dominated total GHG emissions from rice production (73.14%–78.57%), whereas nitrogen fertilizer and diesel were the major sources of indirect emissions (**Figure 6**). Although SRF treatments increased the proportion of fertilizer-related emissions, they substantially reduced the contribution of CH_4_, thereby achieving an overall mitigation effect on GHG emissions.

**Figure 6:**
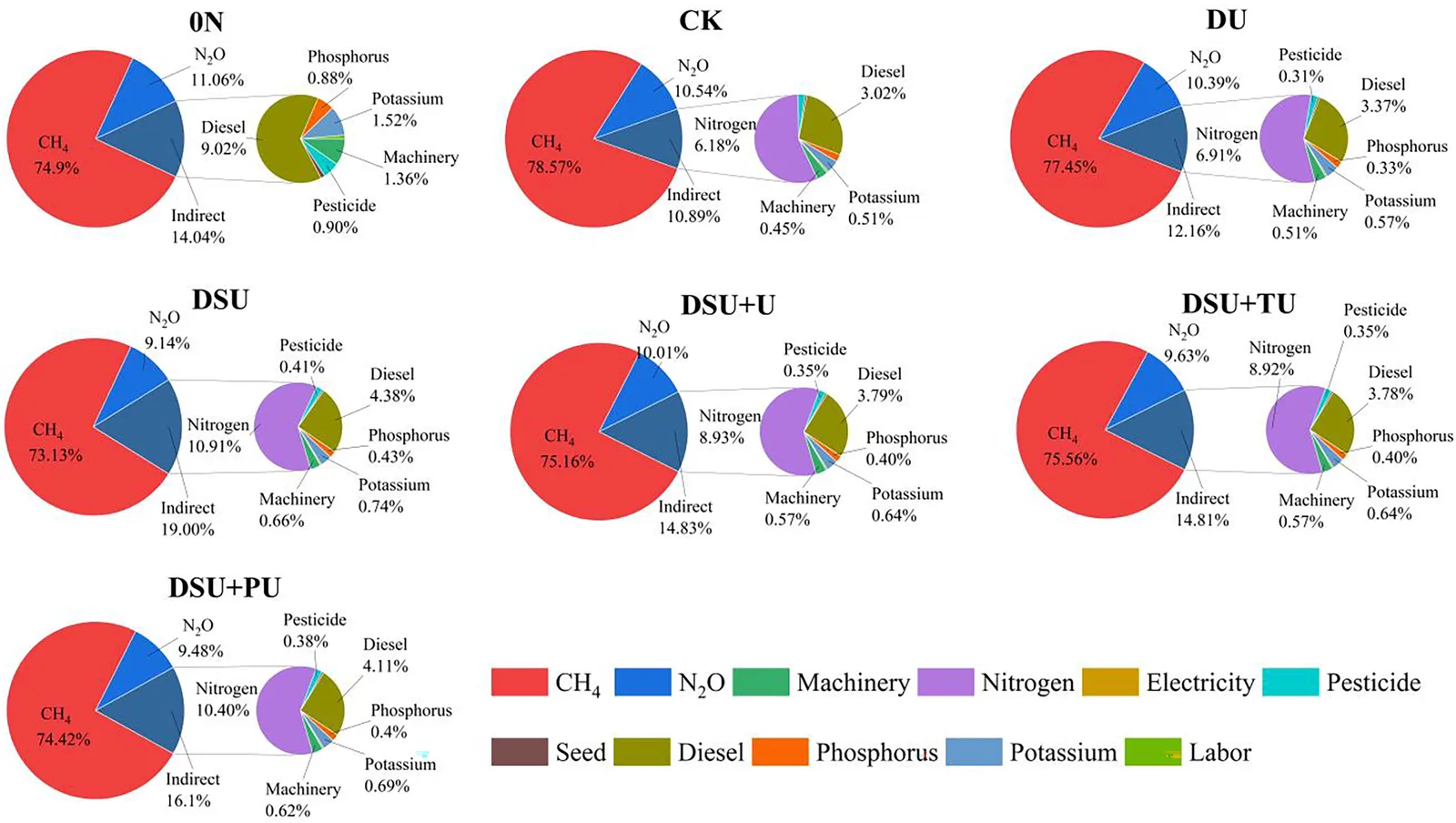
GHG emissions proportion of rice production under different nitrogen fertilizer management modes from 2023 to 2025 (three-year average).

### 3.5 Production cost

From 2023 to 2025, the production cost of rice varied significantly among nitrogen management treatments and were consistently greater than those under the 0N treatment (**Figure 7**). Compared with CK, SRF treatments significantly increased production costs, with a 3-year average rise of 6.02%–9.19%, reaching the highest level under DSU (2496.40–2561.93 US$ ha^-1^). This was largely due to the higher price of slow-release nitrogen fertilizers, which resulted in increased fertilizer expenditure. However, SRF treatments reduced labor-related costs, with a 3-year average decrease of 2.92%–4.38%. In addition, compared with DSU, all combined application treatments significantly reduced total production costs by 2.47%–2.90% due to the substitution of 30% SRF with urea.

**Figure 7:**
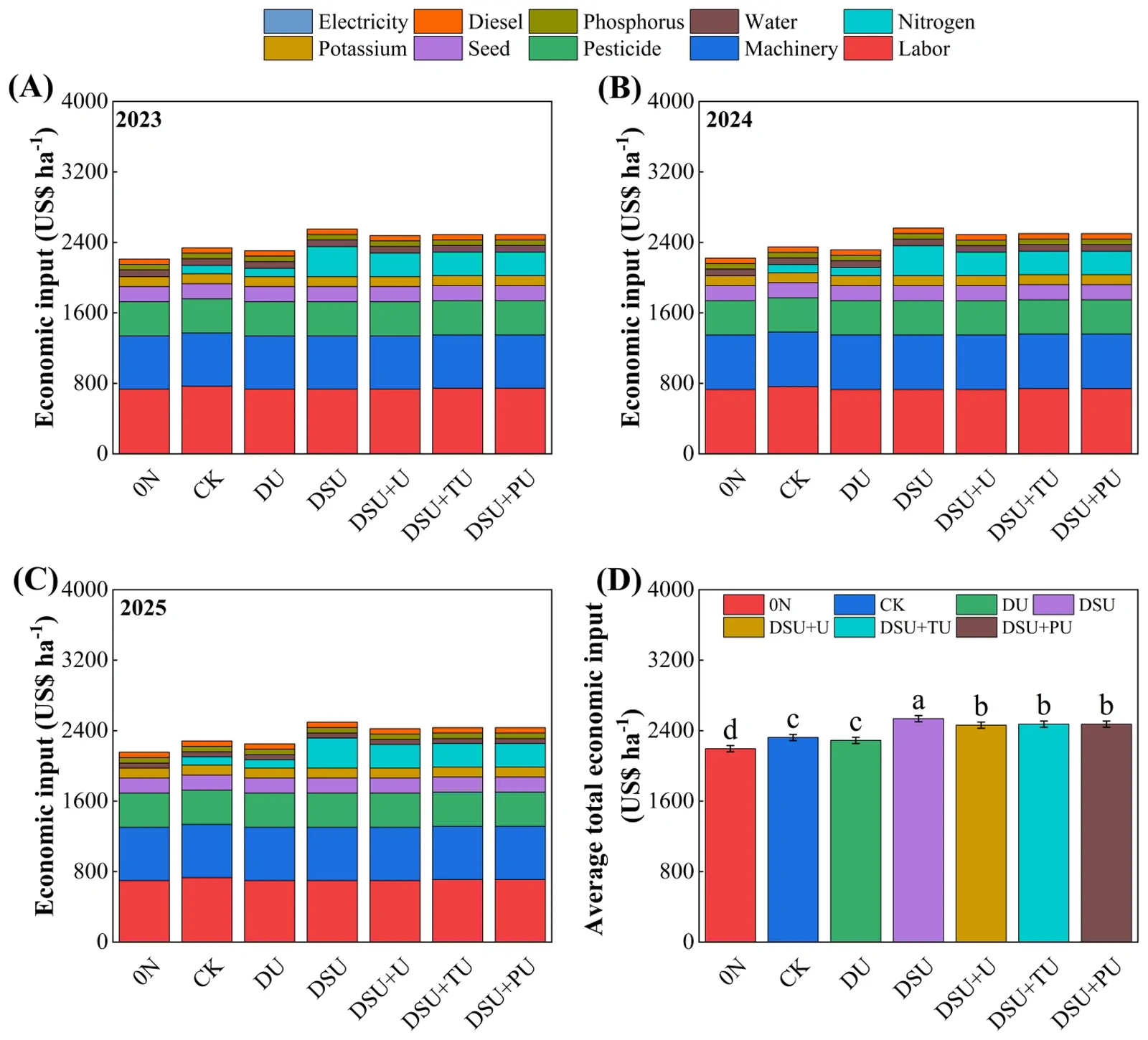
Production cost for rice under different nitrogen fertilizer management modes from 2023 to 2025. Different letters indicate significant differences within the same year at *P* < 0.05. **(A)** Economic input in 2023; **(B)** Economic input in 2024; **(C)** Economic input in 2025; **(D)** Average total economic input from 2023 to 2025.

Across all nitrogen management modes, the relative contribution of each cost component to total production cost followed a consistent order: labor > machinery > pesticides > seeds > potassium fertilizer > nitrogen fertilizer > water > phosphorus fertilizer > diesel > electricity (**Figure 8**). Among them, labor and machinery were the dominant cost components, together accounting for more than 50% of total cost, while nitrogen fertilizer accounted for 4.07%–14.06%. Compared with CK, the proportion of nitrogen fertilizer cost increased dramatically under SRF treatments (by 165.60%–245.45%), with DSU showing the highest value. However, SRF treatments correspondingly reduced the proportion of other production cost items, notably labor cost, which decreased by 8.84%–12.42%.

**Figure 8:**
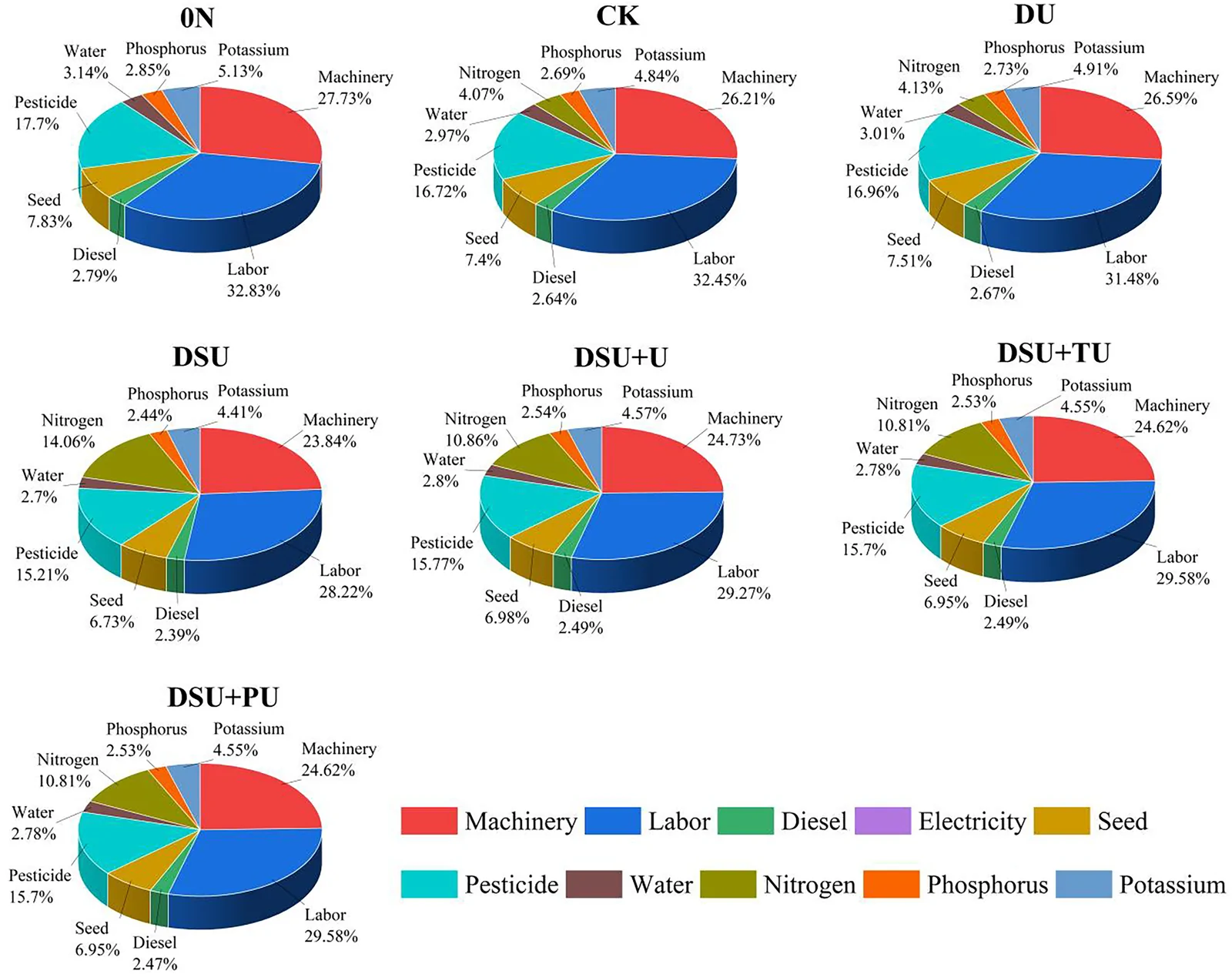
Production cost proportion of rice under different nitrogen fertilizer management modes from 2023 to 2025 (three-year average).

### 3.6 Economic benefits

Different nitrogen management practices had significant effects on the economic benefits of rice production, all of which were markedly higher than those of the 0N treatment (**Figure 9**). Compared with CK, SRF treatments substantially increased gross return, net return and NEEB, with 3-year average increments of 9.64%–23.46%, 10.44%–53.40% and 22.57%–76.78%, respectively, and the greatest improvement was observed under DSU+PU, whereas DU did not differ significantly from CK (**Figures 9A, B, D**). Similarly, SRF treatments enhanced the benefit-to-cost ratio, with urea-supplemented treatments showing a significant increase of 29.51%–44.03% compared with CK, while DSU and CK showed no significant difference (**Figure 9C**). Moreover, relative to DSU, shifting part of the nitrogen supply to later growth stages generally increased gross return, net return, benefit-to-cost ratio and NEEB, with DSU+PU achieving significant improvements of 12.61%, 38.91%, 42.44% and 44.23%, respectively.

**Figure 9:**
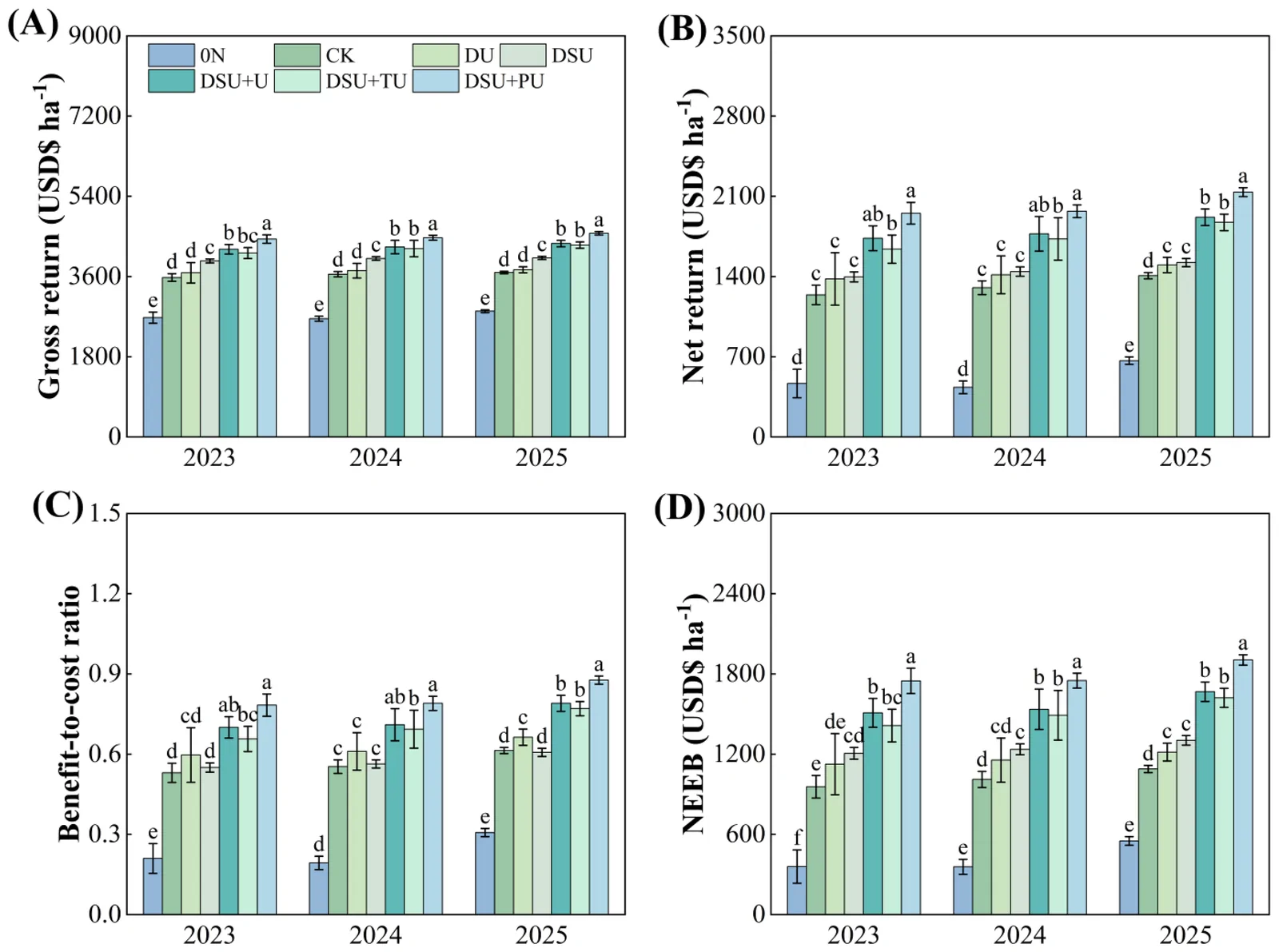
Economic benefits of rice under different nitrogen fertilizer management modes from 2023 to 2025. Different letters indicate significant differences within the same year at *P* < 0.05. **(A)** Gross return; **(B)** Net return; **(C)** Benefit-to-cost ratio; **(D)** NEEB: Net ecosystem economic benefits.

### 3.7 Principal component analysis and comprehensive evaluation

Correlation results indicated a significant positive association between grain yield and NE, NR, BCR, and NEEB, indicating that yield improvement is the core driver of enhanced energy efficiency and increased economic returns. In contrast, CF showed significant positive associations with CH_4_ and N_2_O emissions, while exhibiting significant negative relationships with EUE, EP, and EPF, suggesting that nitrogen management strategies with higher energy efficiency are generally associated with lower yield-scaled environmental costs (**Figure 10A**).

**Figure 10:**
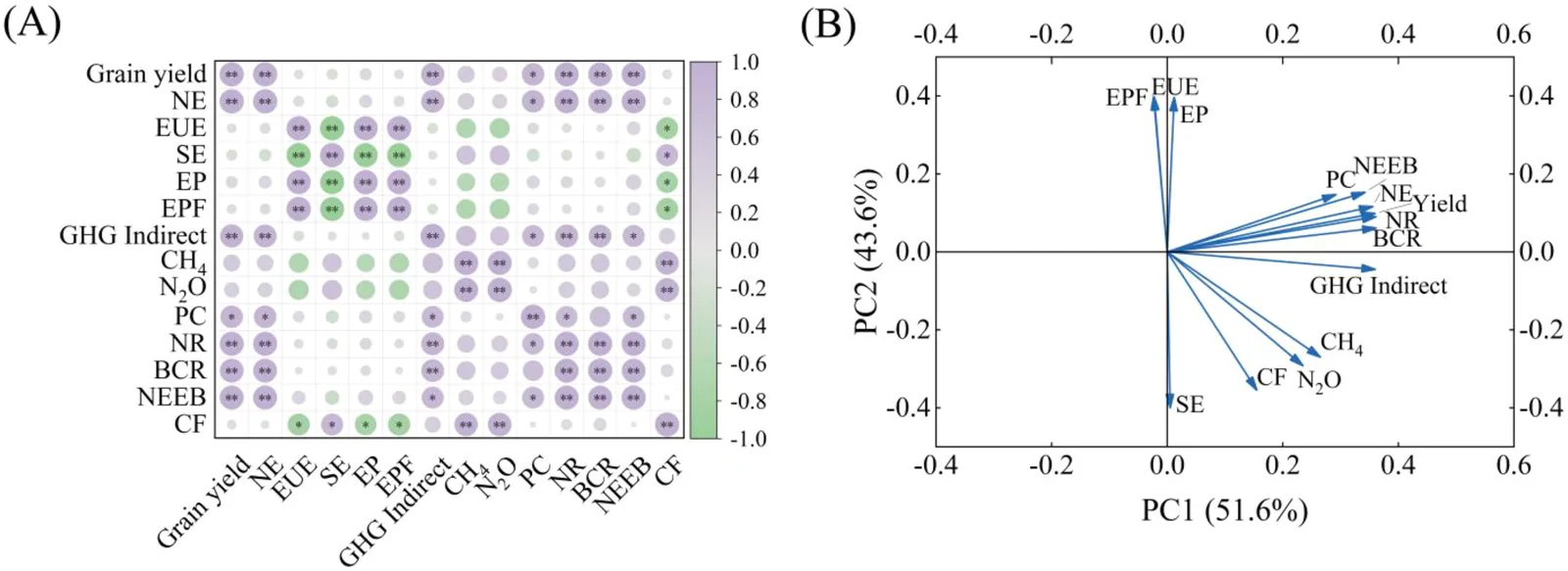
Correlation analysis and principal component analysis. NE: Net energy; EUE: Energy use efficiency; SE: Specific energy; EP: Energy productivity; EPF: Energy profitability; PC: Production cost; NR: Net return; BCR: Benefit-to-cost ratio; NEEB: Net ecosystem economic benefits; CF:Carbon footprint. **(A)** Correlation analysis; **(B)** Principal analysis.

Principal component analysis showed that the first two components together accounted for 95.20% of the total variability. (**Figure 10B**). PC1 (51.60%) was mainly associated with yield, NE, indirect GHG emissions, CH_4_, N_2_O, production cost, net return, benefit-to-cost ratio, and NEEB. PC2 (43.60%) primarily reflected EUE, SE, EP, EPF, and CF. The comprehensive evaluation further showed that, compared with CK, both DU and SRF treatments significantly improved the overall performance score, with DSU+PU achieving the highest score, demonstrating its integrated advantages in boosting yield, enhancing energy efficiency, mitigating greenhouse gas emissions, and improving economic performance (**Table 2**).

**Table 2:** Comprehensive score and ranking.

Treatment	Integrated index value		Subordinate function value		Comprehensive score	Rank
	CI1	CI2	μ(X_1_)	μ(X_2_)	D-value	
0N	-2.17	0.60	0.00	0.80	0.37	7
CK	0.06	-1.63	0.75	0.00	0.41	6
DU	0.05	-1.09	0.75	0.19	0.49	5
DSU	0.13	0.00	0.77	0.58	0.69	4
DSU+U	0.60	0.51	0.93	0.77	0.86	2
DSU+TU	0.54	0.45	0.91	0.74	0.84	3
DSU+PU	0.79	1.16	1.00	1.00	1.00	1
Weight			0.54	0.46		

## 4 Discussion

### 4.1 Analysis of energy inputs, outputs, and efficiency performance under different nitrogen fertilizer management strategies in rice production

Agricultural energy assessment is a key approach for examining the sustainability of cropping systems (Cheng et al., 2023). Across the three study years, average total energy inputs among nitrogen management treatments varied between 27215.38–27404.98 MJ ha^-1^ (**Figure 3**), indicating that rice production in Southwest China is a typical high-energy-input system. This level was comparable to single-season rice in western China (Fu et al., 2024), higher than Nigerian rice systems (Kosemani and Bamgboye, 2020), but lower than irrigated rice in Iran (Pishgar-Komleh et al., 2011). Energy input structure was stable across treatments, with chemical fertilizers contributing over 46% of total input (**Supplementary Figure S2**), consistent with previous reports (Yadav et al., 2017). Nitrogen fertilizer dominated chemical inputs, reflecting a strong dependence on synthetic N (Zhou et al., 2023). Although total energy inputs showed no significant differences among treatments due to identical N rates, SRF treatments showed slightly higher inputs because of greater manufacturing energy, while partial substitution with urea effectively reduced energy input (Hou et al., 2025).

In contrast, energy output differed markedly among treatments and was the main driver of improved energy efficiency. The three-year mean energy output ranged from 261217.79– 321258.16 MJ ha^-1^ (**Figure 4A**), exceeding values reported for rice in central China (Yu et al., 2021). SRF treatments significantly increased grain and straw yields (**Figure 2**), resulting in higher energy output and net energy (**Figures 4A, B**). This may be partly attributed to the combination of slow-release fertilizer and side-deep placement, which likely improves the synchronization between nitrogen supply and crop demand, thereby enhancing nitrogen use efficiency (Liao et al., 2024). In addition, compared with DSU, the DSU+PU treatment showed the greatest increase in energy output, which may be partly attributed to improved synchronization between N supply and crop demand. Although SRF provided sustained N release throughout the growing season (**Figure 1**), its release rate gradually declined during the mid-to-late growth stages. Supplementary urea application at the panicle stage provided a readily available N source during a period of high crop N demand, potentially complementing the declining N release from SRF and thereby promoting yield formation and increasing energy output (Huang et al., 2021).

The three-year average EUE ranged from 9.61–11.76 MJ MJ^-1^ (**Figure 4C**), which is higher than that reported for central China (9.24 MJ MJ^-1^) (Yuan and Peng, 2017). SRF treatments also exceeded the values observed in mechanized rice production systems (11.11 MJ MJ^-1^) (Yang et al., 2022), with DSU+PU achieving the highest EUE. The SE ranged from 2.44–2.99 MJ kg^-1^ (**Figure 4D**), lower than that of smallholder systems (3.19 MJ kg^-1^) (Cheng et al., 2023), and was lowest under DSU+PU, indicating minimal energy consumption per unit grain yield. The EP ranged from 0.33–0.41 kg MJ^-1^ (**Figure 4E**), and the EPF from 8.94–11.09 MJ MJ^-1^ (**Figure 4F**). SRF treatments—particularly DSU+PU—were significantly higher than CK, demonstrating superior energy returns and input–output performance. Overall, side-deep placement of SRF, especially DSU+PU, markedly enhanced energy output and energy-use performance without increasing total energy input, highlighting its strong potential for establishing a high-efficiency, low-consumption rice production system.

### 4.2 Analysis of greenhouse gas emissions and CF under different nitrogen fertilizer management strategies in rice production

CF serves as a key indicator for evaluating the environmental cost and sustainability of agricultural production. In rice systems, total greenhouse gas emissions include both direct field emissions (CH_4_ and N_2_O) and indirect emissions associated with agricultural inputs. CH_4_ dominated total emissions, contributing ~70%, while nitrogen fertilizer and diesel are the main contributors to indirect emissions (**Figure 6**), consistent with previous studies (Fu et al., 2024), highlighting anaerobic paddy soils as a primary mitigation target. Compared with CK, SRF treatments increased indirect GHG emissions due to the higher manufacturing emission coefficients of slow-release fertilizers (**Figures 5A–D**). However, partial substitution with urea significantly reduced indirect emissions relative to DSU, indicating that optimizing fertilizer composition can alleviate upstream carbon emissions (Hou et al., 2025). Nevertheless, SRF treatments may reduce CH_4_ production and mitigate N_2_O emissions by providing a more stable nitrogen supply and regulating soil carbon and nitrogen substrate dynamics, thereby lowering direct greenhouse gas emissions at the field scale (Lan et al., 2024). The results of this study are generally consistent with previous findings, showing that SRF treatments significantly reduced CH_4_ and N_2_O emissions (**Figures 5E–G**). In addition, compared with DSU, the combined application treatments involved surface broadcasting of urea during topdressing, which may increase nitrogen exposure at the soil–atmosphere interface and influence nitrogen transformation processes, potentially resulting in slightly higher CH_4_ and N_2_O emissions. However, the effects of DSU+PU on CH_4_ and N_2_O emissions were relatively small, which may be associated with enhanced plant N uptake at the panicle stage, thereby reducing substrate availability for soil microbial processes (Hu et al., 2025). In addition, greater root activity during this stage may enhance radial oxygen loss and promote CH_4_ oxidation, partly offsetting the increase in CH_4_ emissions associated with urea supplementation (Etesami and Glick, 2024). These mitigation effects largely offset the increased indirect emissions, resulting in an overall reduction in total GHG emissions under SRF treatments (**Figure 5H**). Furthermore, yield-scaled carbon footprint analysis showed that SRF—particularly DSU+PU—significantly reduced CF (**Figure 5I**), indicating a strong synergistic relationship between yield enhancement and emission reduction. Across all nitrogen management strategies, the three-year average CF ranged from 1.10–1.87 kg CO_2_-eq kg^-1^ grain (**Figure 5**), comparable to those reported by Daba et al. (2025), but exceeding the lower range of 0.40–0.75 kg CO_2_-eq kg^-1^ reported by Lan et al. (2024). These discrepancies are likely attributable to differences in system boundary definitions, input structures, emission factors, and rice yield levels. It should be noted that changes in soil organic carbon stocks were not included in the current CF assessment. If sustained SRF application promotes soil organic carbon sequestration, the CF estimated here may therefore be overestimated to some extent, highlighting the need to incorporate soil carbon stock changes into future long-term assessments.

### 4.3 Analysis of production costs and economic benefits of rice under different nitrogen fertilizer management strategies

Production costs and revenues are key determinants of farmers’ adoption of agricultural practices (Lampayan et al., 2015). In this study, total production costs under all nitrogen treatments were significantly greater than those of the 0N treatment, mainly because of the additional expenditure on nitrogen fertilizer. Among nitrogen-applied treatments, CK and DU showed the lowest input costs, whereas SRF treatments exhibited significantly higher total costs because of the high manufacturing cost of slow-release fertilizers (**Figure 7**). However, the DSU+U, DSU+TU, and DSU+PU treatments—where 30% of SRF was replaced with urea—showed markedly lower total costs than DSU, indicating greater flexibility in input configuration. This pattern agrees with observations reported by Wu et al. (2025).

In terms of cost structure, the proportional composition of production costs remained relatively stable across nitrogen management treatments. Labor and machinery consistently accounted for the largest shares—together exceeding 50%—and were the primary contributors to total production cost (**Figure 8**), which is consistent with the typical cost structure of rice production systems in China (Xi et al., 2024). Nitrogen fertilizer costs accounted for 4.07%–14.06% of total production costs across treatments, showing substantial variation. Although SRF treatments increased the share of nitrogen fertilizer cost, the reduced frequency of fertilization operations lowered the relative contributions of machinery and labor inputs. As a result, the overall cost structure became more optimized under SRF treatments.

In terms of economic performance, nitrogen management strategies differed significantly. Compared with CK, SRF treatments markedly increased gross return, net return, and NEEB (**Figure 9**), with DSU+PU achieving the highest economic benefits. These gains were driven by an optimized nitrogen supply that enhanced rice yield while simultaneously reducing carbon and nitrogen losses, thereby improving nitrogen use efficiency and the benefit-to-cost ratio (Wang et al., 2026). Although SRF treatments involved higher fertilizer costs, yield gains were sufficient to offset the additional expenses and substantially improve overall economic returns. Moreover, the significant reductions in CF effectively decreased environmental external costs, resulting in a better trade-off between economic performance and environmental outcomes. A comprehensive multi-dimensional evaluation integrating yield, energy use efficiency, CF and economic performance further demonstrated that, compared with CK, both DU and SRF treatments significantly improved the overall performance score, with DSU+PU ranking highest (**Table 2**). These results demonstrate a clear synergy between high productivity, efficient energy use, lower emissions, and superior profitability in rice systems. These results suggest that combining side-deep applied slow-release nitrogen fertilizer with panicle-stage urea supplementation is not only an economically viable nitrogen management strategy but also a key technological pathway for promoting the transition of rice production systems toward greener, more efficient, and more sustainable development.

However, this study still has certain limitations: (1) The assessment was conducted within a straw-removal production system and did not account for long-term changes in soil organic carbon stocks. Because no rice straw was incorporated into the soil, the observed CH_4_ emissions and treatment responses should be interpreted within this system boundary, as straw incorporation may provide additional organic C substrates for methanogenesis and thereby alter CH_4_ emissions. Moreover, repeated straw removal reduces crop-derived C inputs to the soil and may affect long-term soil C stocks. Therefore, the CF and NEEB results should be interpreted within the straw-removal system evaluated here, and future long-term assessments should incorporate different straw-management practices and changes in soil C stocks. (2) The proportion of urea application was fixed at 30%, and the fertilization timing was determined according to conventional agronomic growth stages. Future studies should explore multiple application ratios and timing combinations to further optimize nitrogen allocation strategies.

## 5 Conclusions

This study comprehensively evaluated the combined impacts of various nitrogen management strategies on rice yield, energy use efficiency, CF and economic performance. The results show that, compared to CK, side-deep placed SRF significantly increased rice yield. While maintaining stable energy input, SRF notably increased energy output, significantly enhancing NE, EUE, EP, and EPF, while effectively reducing SE. Although SRF treatment increased indirect greenhouse gas emissions due to its higher production emissions coefficient, it effectively suppressed CH_4_ and N_2_O emissions, resulting in reduced CF. Additionally, the yield increase effectively offset the higher fertilizer costs, leading to significant improvements in net return, benefit-to-cost ratio, and NEEB. Among all treatments, DSU+PU achieved the highest yield, energy efficiency, and economic returns, while maintaining the lowest carbon footprint, demonstrating superior overall performance. These results highlight that optimized nitrogen management can simultaneously enhance productivity, energy efficiency, and environmental performance.

## Data Availability

The original contributions presented in the study are included in the article/**Supplementary Material**. Further inquiries can be directed to the corresponding authors.
