# Supplementary material for: Side-deep slow-release nitrogen fertilization combined with panicle-stage urea topdressing improves energy efficiency, reduces carbon footprint, and enhances economic performance in rice systems
Source: Front Plant Sci. 2026 Sep 23;17:1962913. doi: 10.3389/fpls.2026.1962913 (PMC13642651; doi:10.3389/fpls.2026.1962913)
Supplement: Supplementary file 1 [file DataSheet1.docx]

Supplementary Material

Supplementary material

**Fig. S1 Meteorological data for the rice season in from 2023 to 2025.**

**Table S1 Basic soil physical and chemical properties.**

| Year | pH | Organic matter  (g kg^-1^) | Total N  (g kg^-1^) | NH_4_^+^-N  (mg kg^-1^) | NO_3_^-^-N  (mg kg^-1^) | Total P  (g kg^-1^) | Total K  (g kg^-1^) | Available P  (mg kg^-1^) | Available K  (mg kg^-1^) |
| --- | --- | --- | --- | --- | --- | --- | --- | --- | --- |
| 2023 | 7.01 | 25.47 | 1.87 | 37.81 | 5.53 | 0.71 | 4.81 | 28.47 | 236.93 |
| 2024 | 6.93 | 24.74 | 1.72 | 33.76 | 5.34 | 0.63 | 5.17 | 21.77 | 226.63 |
| 2025 | 6.90 | 25.22 | 1.81 | 34.54 | 5.46 | 0.68 | 5.02 | 24.24 | 230.14 |

**Table S2 The sowing, transplanting, and harvesting dates of rice every year**

| Year | Sowing (mm/dd) | Transplanting (mm/dd) | Harvest (mm/dd) | Growth duration (d) |
| --- | --- | --- | --- | --- |
| 2023 | 04/03 | 04/25 | 09/02 | 153 |
| 2024 | 03/23 | 04/20 | 08/26 | 157 |
| 2025 | 03/24 | 04/20 | 08/25 | 155 |

**Table S3 Operating procedures and machinery specifications used by farmers.**

| Operating procedures | Production modes |
| --- | --- |
| Sowing in seedbed | Artificial sowing |
| Plowing | 4WD 62.7 kW diesel engine KUBOTA M854K tractor with plow |
| Harrow | 4WD 62.7 kW diesel engine KUBOTA M854K tractor with harrow |
| Seedling transplanting | 4WD 16.1 kW diesel engine KUBOTA SPV-8C25 rice transplanter |
| Irrigation and drainage | Artificial irrigation or drainage by opening/closing the gate of the canal |
| Base fertilizer | KUBOTA FSPV8C25 driven by KUBOTA SPV-8C25 engine and completed synchronously with transplanting |
| Top dressing | Artificial spreading |
| Spraying | 25 W power engine 5.5 kg of MINGHUI sprayer |
| Harvesting and threshing | Artificial harvesting and threshed with 5.5 kW petrol engine CANHONG 5TG-70A half-feed-type threshing machine |

**Table S4 Parameters required to estimate energy inputs when using agricultural machinery.**

| Implements | Unit weight  (kg) | Fuel consumption  (h^−1^) | Operational  life (h) | Field working  efficiency (%) | Power  source |
| --- | --- | --- | --- | --- | --- |
| Plowing machine | 3400 | 16.85 L | 2500 | 65 | Diese |
| Harrow | 3250 | 13.40 L | 2500 | 65 | Diese |
| Rice transplanter | 860 | 3.60 L | 3000 | 60 | Diese |
| Sprayer | 5.5 | 25 W | 250 | 75 | Electricity |
| Harvester | 79 | 2.04 L | 1200 | 60 | Diese |

**Table S5 Energy equivalents of agricultural inputs and outputs and agricultural inputs for rice from sowing to harvesting**

| Items | Unit | Energy equivalent (MJ unit^-1^) | Reference |
| --- | --- | --- | --- |
| **Inputs** |  |  |  |
| Machinery | kg | 68.40 | [1] |
| Labor | h | 1.96 | [2] |
| Diesel | L | 56.31 | [1] |
| Electricity | kWh | 3.60 | [2] |
| Chemical fertilizer |  |  |  |
| SRF (N) | kg | 67.60 | [3] |
| Urea (N) | kg | 66.14 | [2] |
| Phosphorus (P_2_O_5_) | kg | 12.44 | [2] |
| Potassium (K_2_O) | kg | 11.15 | [2] |
| Water | m^3^ | 1.02 | [2] |
| Pesticide |  |  |  |
| Insecticide | kg | 101.20 | [2] |
| Herbicide | kg | 238.00 | [2] |
| Fungicide | kg | 216.00 | [2] |
| Seed | kg | 14.70 | [2] |
| **Outputs** |  |  |  |
| Rice grain | kg | 17.00 | [2] |
| Straw | kg | 12.50 | [2] |

**Table S6 Agricultural inputs for rice from sowing to harvesting under different treatments from 2023 to 2025.**

| Items | Unit | 2023 | | 2024 | | 2025 | |
| --- | --- | --- | --- | --- | --- | --- | --- |
| Machinery |  |  | |  | |  | |
| Plowing machine | h ha^-1^ | 2.10 | | 2.10 | | 2.20 | |
| Harrow | h ha^-1^ | 3.50 | | 3.40 | | 3.40 | |
| Rice transplanter | h ha^-1^ | 3.20 | | 3.10 | | 3.20 | |
| Sprayer | h ha^-1^ | 8.00 | | 8.00 | | 8.00 | |
| Harvester | h ha^-1^ | 34.14 | | 35.50 | | 34.21 | |
| Diesel | L ha^-1^ | 163.45 | | 164.53 | | 166.89 | |
| Electricity | kWh ha^-1^ | 0.20 | | 0.20 | | 0.20 | |
| Water | m^3^ | 3054 | | 2980 | | 2244 | |
| Pesticide |  |  | |  | |  | |
| Insecticide | kg | 2.40 | | 2.40 | | 2.40 | |
| Herbicide | kg | 0.63 | | 0.63 | | 0.63 | |
| Fungicide | kg | 0.44 | | 0.44 | | 0.44 | |
| Seed | kg | 18.00 | | 18.00 | | 18.00 | |
| Phosphorus (P_2_O_5_) | kg | 75.00 | | 75.00 | | 75.00 | |
| Potassium (K_2_O) | kg | 150.00 | | 150.00 | | 150.00 | |
| Labor |  |  | |  | |  | |
| 0N | man-h ha^-1^ | 333.90 | | 331.70 | | 317.50 | |
| CK | man-h ha^-1^ | 348.90 | | 346.70 | | 332.50 | |
| DU | man-h ha^-1^ | 333.90 | | 331.70 | | 317.50 | |
| DSU | man-h ha^-1^ | 333.90 | | 331.70 | | 317.50 | |
| DSU+U | man-h ha^-1^ | 333.90 | | 331.70 | | 317.50 | |
| DSU+TU | man-h ha^-1^ | 338.90 | | 336.70 | | 322.50 | |
| DSU+PU | man-h ha^-1^ | 338.90 | | 336.70 | | 322.50 | |
| Nitrogen |  | SRF (N) | Urea (N) | SRF (N) | Urea (N) | SRF (N) | Urea (N) |
| 0N | kg | 0.00 | 0.00 | 0.00 | 0.00 | 0.00 | 0.00 |
| CK | kg | 0.00 | 150.00 | 0.00 | 150.00 | 0.00 | 150.00 |
| DU | kg | 0.00 | 150.00 | 0.00 | 150.00 | 0.00 | 150.00 |
| DSU | kg | 150.00 | 0.00 | 150.00 | 0.00 | 150.00 | 0.00 |
| DSU+U | kg | 105.00 | 45.00 | 105.00 | 45.00 | 105.00 | 45.00 |
| DSU+TU | kg | 105.00 | 45.00 | 105.00 | 45.00 | 105.00 | 45.00 |
| DSU+PU | kg | 105.00 | 45.00 | 105.00 | 45.00 | 105.00 | 45.00 |

**Table S7 Greenhouse gas emissions and active nitrogen emission coefficients of agricultural inputs for rice production**

| Items | Unit | GHGs emission coefficients  (CO_2_-eq kg unit^-1^) | Reference |
| --- | --- | --- | --- |
| Machinery | kg | 0.071 | [4] |
| Labor | h | 0.036 | [4] |
| Diesel | L | 3.32 | [4] |
| Electricity | kWh | 0.92 | [4] |
| Chemical fertilizer |  |  |  |
| SRF (N) | kg | 9.10 | [3] |
| Urea (N) | kg | 7.48 | [3] |
| Phosphorus (P_2_O_5_) | kg | 0.715 | [3] |
| Potassium (K_2_O) | kg | 0.616 | [3] |
| Pesticide |  |  |  |
| Insecticide | kg | 16.61 | [4] |
| Herbicide | kg | 10.15 | [4] |
| Fungicide | kg | 10.57 | [4] |
| Seed | kg | 0.78 | [4] |

**Table S8 The unit price of rice grains and various inputs in southwestern China is used to evaluate economic benefits.**

| Particulars | Unit | Price (US$ unit^-1^) |
| --- | --- | --- |
| **Inputs** |  |  |
| Machinery |  |  |
| Plowing machine, Harrow, Harvester | h | 13.85 |
| Rice transplanter | h | 16.23 |
| Sprayer | h | 0.12 |
| Diesel | L | 0.95 |
| Electricity | kWh | 0.08 |
| Chemical fertilizer |  |  |
| SRF (N) | kg | 1.00 |
| Urea (N) | kg | 0.29 |
| Phosphorus (P_2_O_5_) | kg | 0.10 |
| Potassium (K_2_O) | kg | 0.45 |
| Labor | h | 2.20 |
| Water | m^3^ | 0.025 |
| Pesticide |  |  |
| Insecticide | kg | 72.26 |
| Herbicide | kg | 120.44 |
| Fungicide | kg | 316.16 |
| Seed | kg | 9.55 |
| **Outputs** |  |  |
| Single-season indica rice | kg | 0.40 |

The data is sourced from the National Development and Reform Commission. The price of slow-release nitrogen fertilizer is the market price.

**Table S9 Analysis of variance for the effects of year, nitrogen management treatments, and their interaction on rice productivity, energy use, carbon footprint, and economic performance.**

| Index | Year（Y) | N treatment (N) | Y×N |
| --- | --- | --- | --- |
| Yield | ** | ** | ns |
| Straw | ** | ** | ** |
| Energy output | ** | ** | ** |
| Net energy | ** | ** | ** |
| EUE | ** | ** | ** |
| SE | ** | ** | ns |
| EP | ** | ** | ns |
| EPF | ** | ** | ** |
| CH_4_ | ** | ** | ** |
| N_2_O | ** | ** | ** |
| TGE | ** | ** | ** |
| CF | ** | ** | ** |
| Gross return | ** | ** | ns |
| Net return | ** | ** | ns |
| BCR | ** | ** | ns |
| NEEB | ** | ** | ns |

NOTE: * and ** indicate significance at P < 0.05 and P < 0.01, respectively; ns indicates no significant difference. EUE: Energy use efficiency, SE: Specific energy, EP: Energy productivity, EPF: Energy profitability, TGE: Total GHG emissions, BCR: Benefit-to-cost ratio.

**Fig. S2 Agricultural energy inputs proportion of rice production under different nitrogen fertilizer management modes from 2023 to 2025 (three-year average).**

**Fig. S3 Dynamics CH_4_ emissions from paddy fields under different N fertilizer management modes. The arrows indicate the timing of basal, tillering, and panicle fertilizer applications.**

**Fig. S4 Dynamics N_2_O emissions from paddy fields under different N fertilizer management modes. The arrows indicate the timing of basal, tillering, and panicle fertilizer applications.**

[1] Yuan. S., Cassman, K., Huang, J., Peng, S., Grassini, P., 2019. Can ratoon cropping improve resource use efficiencies and profitability of rice in central China? Field Crops Res. 234, 66-72.
[2] Yang, Z.Y., Zhu, Y.M., Zhang, X.L., Liao, Q., Fu, H., Cheng, Q.Y., Chen, Z.K., Sun, Y.J., Ma, J., Zhang, J.Y., Li, L.Y., Li, N., 2023. Unmanned aerial vehicle direct seeding or integrated mechanical transplanting, which will be the next step for mechanized rice production in China? —A comparison based on energy use efficiency and economic benefits. Energy, 273, 127223.
[3] Lyu, Y.F., 2022. Impacts of coated controlled release urea application on agronomy - economy - energy consumption - environmental performance of rice production. Doctoral Dissertation of Sichuan Agricultural University.
[4] Lan, C.J., Zou, J.N., Li. J.Y., Xu, H.L., Lin, W.W., Weng, P.Y., Fang, C.X., Zhang, Z.X., Chen, H.F., Lin, W.X., 2024. Slow-release fertilizer deep placement increased rice yield and reduced the ecological and environmental impact in Southeast China: A life-cycle perspective. Field Crops Res. 306, 109224.
